# Magnesium Sulfate as an Adjuvant to Local Anesthetic in Erector Spinae Plane Block: A Systematic Review of Randomized Controlled Trials

**DOI:** 10.3390/life16050726

**Published:** 2026-04-25

**Authors:** Dario Gaetano, Simona Brunetti, Viola Lomonaco, Francesca Piccialli, Angelo Buglione, Umberto Colella, Francesco Coppolino, Vincenzo Pota, Maria Beatrice Passavanti, Pasquale Sansone

**Affiliations:** 1 Department of Women, Child and General and Specialized Surgery, University of Campania “Luigi Vanvitelli”, 80138 Naples, Italy; simona.brunetti@studenti.unicampania.it (S.B.); viola.lomonaco1997@gmail.com (V.L.); francesco.coppolino@unicampania.it (F.C.); vincenzo.pota@unicampania.it (V.P.); mariabeatrice.passavanti@unicampania.it (M.B.P.); pasquale.sansone@unicampania.it (P.S.); 2 School of Medicine and Surgery, University of Campania “Luigi Vanvitelli”, 80138 Naples, Italy; francesca.piccialli@studenti.unicampania.it; 3 Intensive Care Unit, Azienda Ospedaliera di Rilievo Nazionale “San Giuseppe Moscati”, 83100 Avellino, Italy; anbuglione@gmail.com; 4 Anesthesia and Intensive Care Medicine, Department of Critical Care, AORN Ospedali Dei Colli, 80131 Naples, Italy; umberto.colella@ospedalideicolli.it

**Keywords:** erector spinae plane block, magnesium sulfate, adjuvant, postoperative pain, opioid consumption, systematic review, randomized controlled trials, regional anesthesia

## Abstract

**Background:** Magnesium sulfate (MgSO_4_) added to local anesthetics has been investigated as an adjuvant in regional anesthesia, but its role in ultrasound-guided erector spinae plane block (ESPB) remains uncertain. **Methods:** We conducted a PRISMA 2020-compliant systematic review of randomized controlled trials evaluating MgSO_4_ added to the local anesthetic solution in ESPB. In the predefined core comparison (MgSO_4_ added to local anesthetic vs. local anesthetic alone in adult postoperative surgery), four trials (225 participants enrolled; 160 contributing to the comparison) informed the qualitative synthesis. **Results:** Eight randomized controlled trials were included. In the predefined core comparison, 24 h pain intensity was reported heterogeneously and was frequently not extractable as continuous data, precluding pooling. Opioid consumption or rescue analgesia more often favored MgSO_4_; however, outcome metrics, analgesic drugs, and assessment windows were not harmonized, and these effects were not consistently accompanied by reductions in pain intensity at 24 h, limiting their interpretation as true analgesic benefit. Safety reporting was frequently incomplete and often lacked structured adverse event tabulation. Risk of bias varied across domains, and GRADE certainty for all core outcomes was very low. **Conclusions:** Current randomized evidence does not support routine use of MgSO_4_ as an adjuvant in ESPB. Future trials using standardized ESPB techniques, harmonized magnesium dosing strategies, and core outcome sets are required to determine whether magnesium provides clinically meaningful incremental analgesic benefit.

## 1. Introduction

The erector spinae plane block (ESPB) has been widely adopted as part of multimodal analgesia pathways for a range of surgical procedures, including thoracic, breast, abdominal, and spine surgery [1,2]. Although the ESPB is generally regarded as a technically accessible fascial plane block with a favorable safety profile, its clinical effects are variable across settings, and optimization of block duration and quality remains an active area of research [3,4,5]. Procedure-specific and broader meta-analyses have also examined the ESPB across thoracic, breast, vertebral, and mixed postsurgical settings [6,7,8,9,10]. Fascial plane blocks such as the ESPB rely on diffusion of local anesthetic through connective tissue compartments rather than direct perineural deposition, which may contribute to variability in clinical analgesic effects [11].

One commonly explored strategy to improve and prolong regional analgesia is the use of perineural adjuvants added to local anesthetics [12]. However, the choice of adjuvant in the ESPB is not standardized [13]. Trials have evaluated multiple adjuncts—such as alpha-2 agonists, NMDA receptor antagonists, corticosteroids, and anti-inflammatory agents—often using different dosing regimens and outcome definitions [13,14,15]. As a result, evidence synthesis is challenging, and clinicians are left with uncertainty regarding comparative effectiveness and safety [13].

Magnesium sulfate (MgSO_4_) is a biologically plausible adjuvant [16,17]. Analgesic effects have been attributed primarily to modulation of excitatory neurotransmission, including NMDA receptor antagonism and regulation of calcium influx, which may reduce central sensitization [17,18]. Beyond these central mechanisms, magnesium may also exert peripheral effects by modulating calcium-dependent processes involved in neurotransmitter release at nerve terminals, thereby potentially reducing neuronal excitability and enhancing the action of local anesthetics. Experimental studies in animal and ex vivo nerve models have suggested that magnesium may enhance local anesthetic–induced nerve conduction block and interact with voltage-gated ion channels at the peripheral level [19,20]. Magnesium has been evaluated as a systemic analgesic adjunct in perioperative care and chronic pain settings, but its use as an adjuvant to local anesthetics raises additional questions regarding local exposure, diffusion within the fascial plane, and interactions with local anesthetic pharmacodynamics [21,22,23,24]. Magnesium has also been investigated as an adjuvant in neuraxial anesthesia, where systematic reviews have reported heterogeneous effects on analgesic outcomes and highlighted variability in dosing strategies and study designs [25,26]. In the broader regional anesthesia literature, MgSO_4_ has been investigated specifically as an adjuvant to local anesthetics rather than as a stand-alone analgesic strategy. Reviews of peripheral nerve block adjuvants and a meta-analysis focused on perineural magnesium suggest that, when combined with local anesthetics, MgSO_4_ may prolong analgesia and reduce postoperative analgesic requirements in some block settings, although the magnitude and consistency of these effects vary across techniques, local anesthetic regimens, and outcome definitions [12,14,15,21]. Therefore, the potential benefit of MgSO_4_ in the ESPB should be interpreted as a technique-specific question rather than assumed from broader regional anesthesia evidence. In interfascial plane blocks such as the ESPB, these peripheral mechanisms may be particularly relevant given the reliance on drug diffusion within tissue planes rather than direct nerve contact. These uncertainties are compounded by variability in magnesium dosing strategies (fixed-dose versus weight-based), ESPB techniques (level, laterality, and single-level versus bi-level approaches), and perioperative co-analgesia protocols [27,28].

Existing randomized trials investigating magnesium as an ESPB adjuvant span heterogeneous clinical contexts, including different surgical models and comparators (local anesthetic alone, other adjuvants, sham injection, or no-block controls) [28,29]. Outcome reporting also varies, with pain assessed using different scales and timepoints, opioid-related outcomes reported using non-comparable metrics, and safety outcomes inconsistently tabulated [27,30]. These features limit the feasibility and interpretability of quantitative pooling and highlight the need for a structured narrative synthesis that transparently addresses heterogeneity, risk of bias, and certainty of evidence [31].

Therefore, the objective of this systematic review was to synthesize randomized evidence on MgSO_4_ as an adjuvant added to local anesthetics in an ultrasound-guided ESPB. We aimed (i) to evaluate postoperative pain intensity, opioid consumption/rescue analgesia, and safety outcomes; (ii) to describe protocol variability in magnesium dosing and ESPB technique; (iii) to contextualize magnesium’s effects relative to alternative adjuvants; and (iv) to assess risk of bias and certainty of evidence using RoB 2 and GRADE [32,33]. A predefined core comparison (magnesium plus local anesthetic versus local anesthetic alone in adult postoperative surgery) and an anchor pain outcome at 24 h were used to enhance interpretability.

## 2. Materials and Methods

### 2.1. Protocol and Reporting Standards

This systematic review was designed to identify, select, and synthesize evidence from randomized controlled trials evaluating magnesium sulfate as an adjuvant in ultrasound-guided erector spinae plane block. It was conducted in accordance with the Preferred Reporting Items for Systematic Reviews and Meta-Analyses (PRISMA 2020) statement and followed methodological guidance from the Cochrane Handbook for Systematic Reviews of Interventions [31,34,35]. The review was conducted according to a predefined protocol. The PICO framework and full search strategies are provided in the Supplementary Materials (Supplementary Table S1). The search strategy was developed by the authors based on prior experience in systematic reviews. The protocol has been registered in PROSPERO (CRD420261343098). No amendments to the registered protocol were made. No quantitative meta-analysis was prespecified due to anticipated heterogeneity in surgical settings, magnesium dosing strategies, outcome definitions, and reporting formats. The PRISMA 2020 checklist is provided in Supplementary Table S2. The review was conducted between December 2025 and February 2026, with the final literature search performed on 8 February 2026. Ethics committee approval was not required for this systematic review using only publicly available data from published studies.

### 2.2. Eligibility Criteria

Studies were eligible if they met the following criteria: (1) randomized controlled trials; (2) adult or pediatric patients receiving an ultrasound-guided erector spinae plane block (ESPB); (3) MgSO_4_ used as an adjuvant added to the local anesthetic solution for ESPB injection; and (4) reporting at least one clinically relevant outcome (pain scores, opioid consumption, rescue analgesia, or safety outcomes). Trials comparing magnesium with local anesthetic alone, other adjuvants, sham injections, or no-block controls were eligible. Non-randomized studies, observational designs, case reports, editorials, narrative reviews, animal studies, and conference abstracts without full data were excluded. Trial registry records without corresponding peer-reviewed full-text publications were excluded. If non-peer-reviewed reports had met eligibility criteria, they would have been considered at higher risk of bias and included only in qualitative synthesis. Eligibility criteria were defined a priori according to the review question and PICO framework, consistent with PRISMA 2020 and Cochrane methodological guidance [31,34,35].

### 2.3. Information Sources and Search Strategy

**Figure 1:**
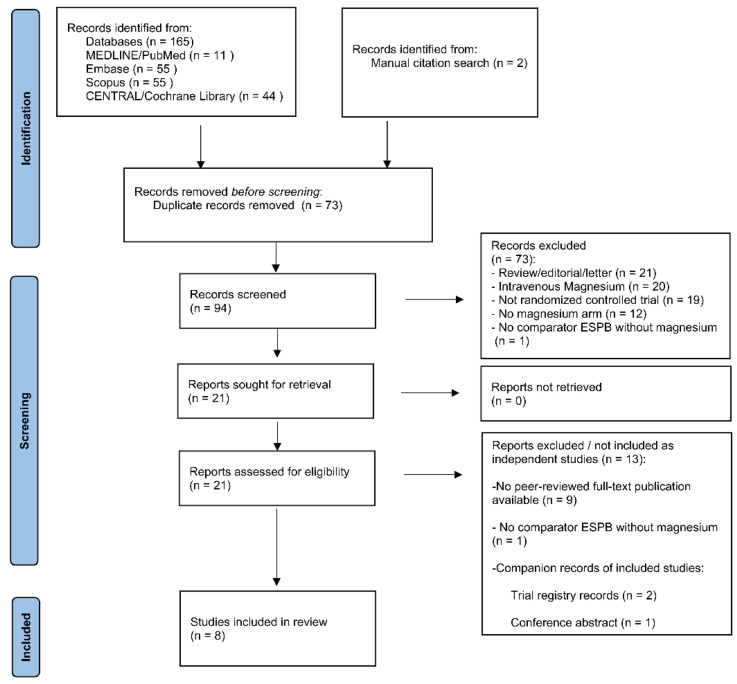
PRISMA 2020 flow diagram of the study selection process.

A comprehensive literature search was performed to identify randomized controlled trials evaluating magnesium sulfate as an adjuvant to local anesthetics in an ultrasound-guided erector spinae plane block (ESPB). The study selection process followed the PRISMA 2020 framework, including duplicate removal, title/abstract screening, and full-text assessment, as summarized in the PRISMA flow diagram (Figure 1) [31,34,36]. The electronic databases MEDLINE (via PubMed), Embase, Scopus, and CENTRAL were searched from their inception (i.e., from the earliest records available in each database) to 8 February 2026. Search terms combined controlled vocabulary and free-text terms related to ‘erector spinae plane block’, ‘magnesium’, and ‘adjuvant’. No language restrictions were applied at the search stage. In addition, the reference lists of relevant articles and reviews were manually screened to identify additional eligible studies. No separate formal gray literature search was conducted; however, non-peer-reviewed records retrieved through database searches or manual citation tracking were screened and assessed for eligibility according to the predefined criteria. Trial registry records were, therefore, included when identified through these processes but were not searched as a dedicated gray literature source. Companion records (e.g., trial registry entries or conference abstracts) were identified during the selection process, linked to their corresponding primary studies, and not considered as independent units of analysis. The full electronic search strategies for all databases are provided in Supplementary Table S1.

### 2.4. Study Selection Process

After removal of duplicate records, two pairs of independent reviewers (D.G. and S.B., and V.L. and U.C.) screened titles and abstracts against predefined eligibility criteria. Potentially relevant studies were retrieved in full text and independently assessed for eligibility by the same reviewers. Reasons for exclusion at the full-text stage were recorded. Full-text records assessed for eligibility, including companion records of included studies, are reported in Supplementary Table S3, together with their classification and the primary reason for non-inclusion as independent studies. When multiple exclusion criteria were applied, records were assigned a primary reason for exclusion. Disagreements were resolved through discussion and consensus; when necessary, a senior reviewer (P.S.) was consulted to reach a final decision. Independent duplicate screening and documentation of exclusions were conducted in line with standard systematic review methodology [31,34,35]. The study selection process is summarized in the PRISMA flow diagram (Figure 1).

### 2.5. Data Extraction

Data extraction was performed independently by two reviewers (D.G. and S.B.) using a predefined structured extraction form. Extracted data included study design, clinical setting, sample size, ESPB technique (level, laterality, dose of MgSO_4_), comparator arms, pain scale and timepoints, opioid consumption metrics, and rescue analgesia criteria, including their definitions (e.g., rescue thresholds and administration protocols), which were interpreted as indirect measures of analgesic effect potentially influenced by protocol-dependent factors and reported adverse events. Discrepancies between reviewers were resolved through discussion and consensus; when necessary, a senior reviewer (P.S.) was consulted to reach a final decision. When outcome data were missing, incompletely reported, or not extractable (e.g., reported only graphically without tabulated numerical values), no imputation was performed, and the study authors were not contacted, in line with the predefined decision to restrict the analysis to published data. Only data available in the published full-text articles were considered for analysis. Non-extractable outcomes were explicitly documented and described narratively. Data extraction followed standard systematic review practice, including independent duplicate extraction and conservative handling of non-extractable data. A predefined standardized data extraction form was used to ensure consistency across studies [31,34,35].

### 2.6. Risk of Bias Assessment

Risk of bias in included studies was assessed to evaluate the internal validity of the included trials and to inform the interpretation of the findings and the GRADE assessment of the certainty of evidence. The Cochrane Risk of Bias 2 (RoB 2) tool for randomized controlled trials was used to assess bias across the standard domains [28]. Two reviewers (D.G., S.B.) independently performed the assessment. Inter-reviewer disagreements were resolved by discussion; when necessary, a third reviewer (P.S.) was consulted. Assessments were performed outcome-specifically for pain intensity and opioid/rescue outcomes. The following domains were examined: bias arising from the randomization process; deviations from intended interventions; missing outcome data; measurement of the outcome; and selection of the reported result. Each domain and overall judgments were categorized as low risk of bias, some concerns, or high risk of bias. Safety outcomes were evaluated descriptively due to inconsistent reporting and low event counts across trials.

### 2.7. Synthesis Methods

Given substantial clinical and methodological heterogeneity across trials, the results were synthesized narratively according to structured synthesis principles. A predefined core comparison (MgSO_4_ plus local anesthetic versus local anesthetic alone in adult postoperative surgery) was established to enhance interpretability. The anchor outcome for pain was 24 h postoperative pain intensity.

When continuous data were unavailable or inconsistently reported (e.g., dichotomized categories or figure-only presentation without extractable summary statistics), the direction of effect was described qualitatively. Due to heterogeneity in surgical settings, dosing strategies, comparator arms, outcome definitions, and reporting formats, no quantitative meta-analysis was performed. In addition, several trials reported outcomes using non-extractable formats (e.g., graphical reporting without tabulated summary statistics), preventing reliable calculation of pooled effect estimates.

### 2.8. Assessment of Certainty of Evidence

Certainty of evidence for predefined core subgroup outcomes was assessed using the Grading of Recommendations Assessment, Development, and Evaluation (GRADE) framework [33]. For each outcome, the body of evidence was evaluated across domains that may decrease certainty, including risk of bias (study limitations), inconsistency (unexplained heterogeneity across results), indirectness (applicability to the review question), and imprecision (confidence in effect estimation). Although a subgroup of trials comparing magnesium sulfate plus local anesthetic versus local anesthetic alone in adult postoperative surgery was identified, heterogeneity in surgical procedures, dosing regimens, and outcome reporting precluded a meaningful quantitative synthesis.

Certainty could be upgraded in the presence of large effect sizes, dose–response gradients, or residual confounding; however, no upgrading factors were identified in this review. Summary of findings tables were generated using GRADEpro GDT software (web-based software; McMaster University and Evidence Prime, Kraków, Poland; available at https://www.gradepro.org, accessed on 22 April 2026).

### 2.9. Standardized Exclusion Categories for the PRISMA Flow Diagram

To enhance transparency and consistency in reporting, predefined exclusion categories were applied during full-text screening. These included non-randomized designs; absence of magnesium added to the ESPB local anesthetic solution; magnesium administered systemically rather than as an ESPB adjuvant; absence of an ESPB; ineligible populations relative to the predefined review question; comparator arms not relevant to the review objective; absence of extractable clinical outcomes; conference abstracts without full-text availability; duplicate publications; protocols without results; and animal or cadaveric studies. Companion records (e.g., trial registry entries or conference abstracts corresponding to included studies) were retained for transparency and were not considered independent studies.

## 3. Results

### 3.1. Study Selection

The study selection process is summarized in the PRISMA flow diagram (Figure 1). Database searching identified 165 records, and manual citation searching identified two additional records (total 167). After removal of duplicates (73), 94 records were screened by title/abstract, and 73 were excluded: review/editorial/letter (primary reason), *n* = 21; intra-venous (IV) magnesium (primary reason), *n* = 20; not randomized controlled trial (primary reason), *n* = 19; no magnesium arm (primary reason), *n* = 12; and no comparator ESPB without magnesium (primary reason), *n* = 1. Twenty-one full-text records were assessed for eligibility. Of these, 13 were not included as independent studies: 10 were excluded from full-text records, and three were companion records of included studies. Among the excluded full-text records, the primary reasons were absence of a peer-reviewed full-text publication (*n* = 9) and no comparator ESPB without magnesium (*n* = 1). Details are provided in Supplementary Table S3. Eight studies were included in the qualitative synthesis [27,28,29,30,37,38,39,40].

### 3.2. Study Characteristics

Eight randomized trials were included (Table 1). Trials covered heterogeneous clinical contexts, including adult postoperative surgical pathways and indirect contexts (pediatric surgery and chronic neuropathic pain) [29,30]. Surgical settings included spine procedures, breast surgery, and laparoscopic abdominal surgery; one trial enrolled pediatric patients undergoing inguinal hernia repair, and one trial evaluated the ESPB in postherpetic neuralgia. Comparators varied and included local anesthetic alone, other perineural adjuvants, sham injection, and no-ESPB controls [28,29].

Intervention protocols were also heterogeneous. MgSO_4_ dosing was delivered using fixed-dose and weight-based strategies and was administered as part of different injectate compositions and volumes [27,37]. ESPB technique varied across studies with respect to block level(s) (single-level versus multi-level approaches) and laterality (unilateral versus bilateral blocks) [37,39]. Co-analgesic pathways and rescue analgesia criteria were variably described [30,39]. No study reported pharmacokinetic data on local magnesium concentrations, plasma magnesium changes, cerebrospinal fluid penetration, or surrogate biomarkers of NMDA modulation, and no dose–response relationship was formally evaluated across trials. These features informed the decision to prioritize structured narrative synthesis and to predefine a core comparison to enhance interpretability. Table 1 summarizes study characteristics and the outcomes reported by the included trials; it is intended to describe study design and reporting patterns.

### 3.3. Risk of Bias in Included Studies

The risk of bias results for pain and opioid/rescue outcomes, assessed using the RoB 2 tool, are summarized in Table 2. Overall, most trials raised some concerns due to incomplete reporting of the randomization process (e.g., allocation concealment not clearly described), limited detail on blinding procedures, and/or outcome reporting limitations. Concerns regarding the selection of the reported result were common when prespecified analysis plans were not clearly accessible or when outcomes were presented in formats that limited extraction (e.g., dichotomized reporting or figure-only presentation) [37,38]. Missing outcome data were generally limited; however, only a subset of trials reported explicit adverse event counts, while others provided incomplete or qualitative safety data, and safety reporting frequently lacked tabulated incidence counts, restricting confidence in harm assessment [27,30,38,39]. Domain-level RoB 2 judgments by outcome are provided in Supplementary Table S4.

### 3.4. Core Subgroup: MgSO_4_ Plus Local Anesthetic Versus Local Anesthetic Alone in Adult Postoperative Surgery

**Figure 2:**
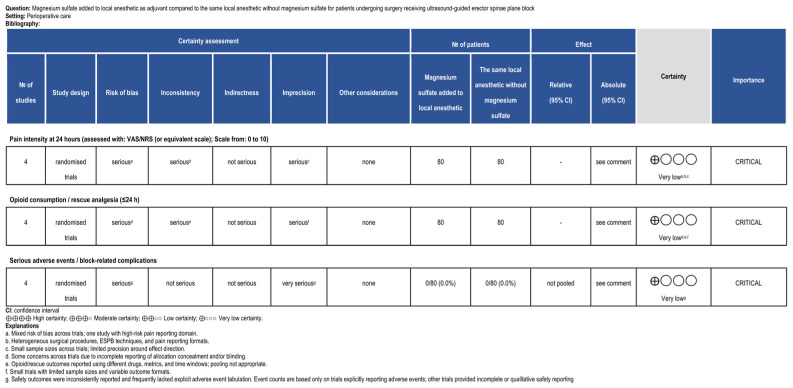
Summary of findings (GRADE) for the predefined core comparison (magnesium sulfate added to local anesthetic as adjuvant versus local anesthetic alone in adult postoperative surgery). Certainty of evidence was rated as very low across all outcomes due to risk of bias, inconsistency, and imprecision.

Four adult postoperative trials included a local anesthetic-only ESPB control arm and formed the predefined core subgroup for qualitative synthesis (*n* = 225) [27,37,38,40]. Table 3 summarizes the review outcomes for this predefined core comparison, specifically pain at 24 h and opioid/rescue analgesia outcomes. Across the four RCTs, 225 participants were enrolled; 160 contributed to the magnesium versus local anesthetic alone core comparison, as three trials had a third active comparator arm. The anchor pain outcome was pain intensity at 24 h. Across core outcomes, certainty of evidence was rated very low according to GRADE due to risk of bias concerns, inconsistency in reporting formats and clinical contexts, and imprecision related to small sample sizes (Figure 2).

Pain at 24 h was reported heterogeneously. Two trials provided extractable numerical 24 h pain values as median (range) and did not show a clear between-group difference at 24 h (rest and/or movement where reported) [27,40]. One trial reported pain only in dichotomized categories at 24 h, and one trial presented 24 h pain exclusively as boxplots without tabulated summary statistics, precluding numerical extraction [37,38]. Given the variability in reporting formats, no pooling was performed for pain outcomes.

Opioid-related outcomes in the core subgroup generally favored magnesium but used non-comparable metrics. Two trials reported continuous 24 h opioid totals (mean ± SD), and both showed lower opioid requirements with magnesium [27,40]. One trial reported rescue analgesia as an event-based outcome (patients receiving tramadol 50 mg), with fewer rescue events in the magnesium group [38]. One trial reported total morphine consumption over 48 h and was used for direction of effect only [37].

Safety reporting was inconsistent across the core subgroup. Two trials provided explicit adverse event counts (e.g., postoperative nausea/vomiting and pneumothorax), with similar rates between groups and no pneumothorax reported [37,40]. The remaining trials primarily reported hemodynamic summaries and/or qualitative statements without extractable event counts [27,38]. Non-serious adverse events (e.g., postoperative nausea and vomiting) were variably reported across trials and could not be consistently attributed to the ESPB intervention itself [37,40]. These outcomes were, therefore, described narratively but were not included in the core safety synthesis. Certainty of evidence for core outcomes was rated very low (Figure 2).

### 3.5. MgSO_4_ Versus Other Adjuvants in Adult Postoperative Surgery

Three adult postoperative trials compared MgSO_4_ with alternative adjuvants added to local anesthetic [37,39,40]. These studies primarily inform comparative effectiveness between adjuvant strategies rather than magnesium’s incremental effect over local anesthetics alone. Across trials, differences in comparator choice, magnesium dosing, ESPB technique, and outcome definitions limited cross-study interpretability; therefore, the results were synthesized narratively as contextual evidence for adjuvant selection and protocol variability.

### 3.6. Three-Arm Scoliosis Trial MgSO_4_ Versus Dexmedetomidine Versus No-ESPB Control

One three-arm randomized trial in corrective scoliosis surgery compared MgSO_4_ with dexmedetomidine as adjuvants in a bilateral bi-level ESPB and included a no-ESPB control arm [28]. Pain was summarized as a time-weighted average over 0–48 h, and opioid consumption was reported over 48 h. Because a local anesthetic-only ESPB arm was not included, the incremental effect of magnesium over local anesthetic alone could not be isolated; the trial was incorporated as contextual evidence regarding adjuvant choice and protocol variability.

### 3.7. Magnesium Sulfate Versus Dexmedetomidine in Lumbar Spine Surgery

One randomized controlled trial evaluated an ultrasound-guided bilateral ESPB with bupivacaine–magnesium sulfate versus bupivacaine–dexmedetomidine in patients undergoing lumbar spine surgery [39]. This single-blind study included 52 adult patients randomized to receive either magnesium sulfate (125 mg added to 0.25% bupivacaine per side) or dexmedetomidine (1 µg/kg added to bupivacaine). The block was performed at the T10 level under ultrasound guidance.

Postoperative pain intensity (VAS) was assessed at multiple time points (2–12 h), and nalbuphine was used as rescue analgesia. The dexmedetomidine group demonstrated significantly lower VAS scores at most postoperative time points and reduced rescue analgesic requirements compared with the magnesium group.

Given that this comparison does not include a local anesthetic-alone control group, it was not incorporated into the core subgroup analysis (magnesium plus local anesthetic versus local anesthetic alone) nor into the corresponding GRADE certainty assessment. However, it contributes to the qualitative synthesis addressing head-to-head comparisons between magnesium and other single perineural adjuvants within the ESPB.

### 3.8. Indirect Clinical Contexts of Pediatric Surgery and Postherpetic Neuralgia

One pediatric trial in inguinal hernia repair and one non-surgical trial in postherpetic neuralgia were analyzed as indirect evidence streams rather than contributing to the core subgroup analysis [29,30]. These contexts differ substantially from adult postoperative surgical analgesia in baseline pain trajectories, co-analgesic pathways, and outcome selection. Accordingly, these studies were not used to draw inferences about routine perineural magnesium use in an adult postoperative ESPB but were included to describe the breadth of clinical evaluation and reporting practices.

### 3.9. Protocol Variability Analysis Across All Included Trials

Protocol heterogeneity is summarized in Table 4 and in greater detail in Supplementary Table S5 (Protocol Variability Matrix). Because Table 4 is intended to summarize protocol heterogeneity rather than clinical outcomes, it is presented as contextual evidence to support the interpretation of between-study variability.

Across the eight included trials, heterogeneity was observed at multiple levels. Intervention protocols varied in magnesium dosing strategy (fixed versus weight-based), injectate composition and volume, and the presence of concomitant adjuvants or multimodal analgesic pathways [27,37]. Technique heterogeneity included differences in ESPB level selection and laterality and in whether a single-level or multi-level approach was used [37,39]. Comparator heterogeneity included a local anesthetic-only ESPB, alternative adjuvants, sham injections, and no-ESPB controls [28,29]. No consistent dose–response relationship was observed across studies. The absence of pharmacokinetic data and of a clear dose–response relationship across studies limits mechanistic interpretation and raises uncertainty regarding the biological plausibility of a direct local analgesic effect. Outcome heterogeneity was prominent. Pain outcomes were assessed using different scales and timepoints and were variably reported as continuous measures, dichotomized categories, or graphics without tabulated values; opioid outcomes were reported using different drugs, windows, and rescue definitions; and safety reporting was frequently incomplete [37,38]. This combined heterogeneity limited the feasibility and interpretability of quantitative pooling and supports the need for standardized protocols and harmonized outcome reporting in future trials. Heterogeneity was considered primarily clinical and methodological rather than statistical, precluding meaningful quantitative synthesis.

### 3.10. Certainty of Evidence

Certainty of evidence for the predefined core subgroup outcomes (pain at 24 h, opioid consumption/rescue analgesia, and serious block-related adverse events) was rated as very low using the GRADE framework (Figure 2). Non-serious adverse events (e.g., postoperative nausea and vomiting) were variably reported and, therefore, described narratively rather than incorporated into the GRADE summary; only trials explicitly reporting adverse event counts contributed to the GRADE analysis. Therefore, denominators reflect only studies with extractable safety data. Downgrading reflected cumulative concerns related to study limitations, heterogeneity across settings and reporting formats, and imprecision from small sample sizes.

## 4. Discussion

### 4.1. Principal Findings and Clinical Interpretation

This review demonstrates that the current randomized evidence base evaluating MgSO_4_ as an adjuvant added to local anesthetic in the ESPB is small, heterogeneous, and methodologically inconsistent. The limited number of trials reflects the early stage of investigation of magnesium as an ESPB adjuvant. In the predefined core comparison (magnesium plus local anesthetic versus local anesthetic alone in adult postoperative surgery), 24 h pain outcomes were inconsistently reported and frequently non-extractable as continuous data. Opioid-related outcomes more frequently favored magnesium; however, these were reported using heterogeneous metrics and non-harmonized time windows. Importantly, opioid-sparing effects observed in the absence of consistent reductions in pain intensity should be interpreted cautiously. Pain intensity represents a direct patient-centered measure of analgesia, whereas opioid consumption is an indirect outcome that may be influenced by study protocols and contextual factors. This distinction between direct and indirect measures of analgesic efficacy is recognized in methodological recommendations for pain trials [41]. Adverse event reporting was inconsistent and often lacked incidence counts. Collectively, these limitations preclude reliable quantitative effect estimation. These findings must be interpreted in light of the very low certainty of evidence across all core outcomes, which limits confidence in both effect magnitude and reproducibility (Figure 2). They should also be interpreted in the context of the broader ESPB literature. Recent systematic reviews and meta-analyses have generally supported the analgesic efficacy of the ESPB across several surgical settings, although effect sizes vary according to procedure type, technique, and outcome definitions [2,6,7,8,9,10]. In contrast, the present review addressed a narrower and more specific question: whether magnesium provides incremental benefit when added to local anesthetics in the ESPB. We found that the currently available randomized evidence is too heterogeneous and methodologically inconsistent to support a clear clinical advantage.

### 4.2. The Standardization Gap in Magnesium as an Adjuvant in the ESPB

A central finding of this review is the marked absence of protocol standardization across trials. Magnesium dosing strategies varied substantially (fixed-dose versus weight-based), injectate volumes were inconsistent, and ESPB techniques differed in block level, laterality, and single-level versus bi-level approaches. Pain outcomes were assessed using different scales and analytic formats, including continuous measures, dichotomized categories, and figure-only reporting. Opioid outcomes were not consistently converted into morphine milligram equivalents, and rescue criteria were variably defined. Such variability prevents identification of a reproducible dose–response relationship and limits external validity. Previous systematic reviews on magnesium as a perineural adjuvant of local anesthetics have reported substantial variability in magnesium dosing strategies and the absence of a consistent dose–response relationship [21,42]. However, the present analysis further highlights that the lack of pharmacokinetic data and the relatively low doses used raise additional uncertainty regarding the biological plausibility of a direct local analgesic effect. In addition, none of the included trials measured local magnesium concentrations, plasma magnesium changes, surrogate biomarkers consistent with NMDA pathway modulation, or cumulative exposure, including possible concomitant systemic magnesium administration, nor did they quantify local or systemic drug concentrations following interfascial injection. These gaps further limit the ability to define a reliable safety profile and preclude any assessment of dose–exposure relationships. Accordingly, any mechanistic explanation for magnesium in the ESPB should be regarded as hypothetical rather than demonstrated in the current clinical evidence base. In its current state, the literature does not yet provide a sufficiently standardized framework to support routine use of magnesium as an adjuvant in the ESPB. Consequently, the current evidence base remains exploratory rather than confirmatory with respect to magnesium’s role as an ESPB adjuvant. The field appears to be experimenting with magnesium under widely variable conditions rather than evaluating a harmonized intervention strategy. This lack of standardization contrasts with the broader literature on peripheral nerve block adjuvants, in which heterogeneity has also been recognized but where more consolidated patterns of use and outcome reporting are available than in ESPB-specific magnesium studies [12,13,14,15].

### 4.3. Comparator Landscape and Incremental Effect Uncertainty

The comparator landscape further complicates interpretation. Trials have compared magnesium with local anesthetic alone, alpha-2 agonists, NMDA antagonists, non-steroidal anti-inflammatory agents, corticosteroids, sham injections, and no-block controls [28,29,30,37,40]. Multi-arm trials lacking a local anesthetic-only ESPB arm cannot isolate magnesium’s incremental effect over local anesthetic alone [28,39]. As a result, current evidence does not clarify whether magnesium provides an additive benefit or how it performs relative to alternative adjuvants under standardized conditions. This uncertainty is particularly relevant because meta-analyses in peripheral nerve block settings have suggested that perineural magnesium may prolong analgesia and reduce postoperative analgesic requirements, while also highlighting substantial heterogeneity across block types, dosing strategies, outcome definitions, and reproducibility of effects [21,42,43]. Moreover, even within conventional regional anesthesia techniques targeting more defined neural structures, the reported benefits of magnesium are not uniform across outcomes [44,45,46]. While several randomized trials have reported prolongation of sensory block duration and reductions in early postoperative pain, these findings have not been uniformly accompanied by consistent reductions in opioid consumption or clearly clinically meaningful differences in patient-centered outcomes [44,45,46]. The current ESPB-specific evidence does not reproduce these effects with sufficient consistency, supporting the need for technique-specific evaluation rather than extrapolation from other regional anesthesia settings. This discrepancy may reflect fundamental differences between conventional peripheral nerve blocks and fascial plane blocks such as the ESPB, including variability in anatomical targets and injectate spread, which may attenuate or render less predictable any incremental effect of magnesium [11].

### 4.4. Safety Reporting and Under-Ascertainment of Adverse Events

No clear signal of increased serious adverse events attributable to magnesium used as an adjuvant in the ESPB was identified; however, safety reporting was frequently incomplete. Several trials did not tabulate adverse event counts, did not predefine safety endpoints, or had limited reporting to hemodynamic summaries [27,39]. The absence of structured neurologic follow-up, standardized definitions of block-related complications, and explicit incidence reporting limits confidence in safety conclusions, particularly for rare events. Importantly, magnesium cannot be considered pharmacologically inert. Systemic administration is associated with dose-dependent effects, including hypotension, bradycardia, and neuromuscular modulation [16,23]; available evidence from neuraxial administration suggests no clear signal of neurotoxicity in clinical studies; however, safety data remain limited and insufficient to exclude potential adverse neural effects [25,26,47]. Preclinical studies have reported dose-dependent neurodegenerative changes following intrathecal magnesium administration, particularly at higher concentrations or with repeated exposure [48,49]. In the context of regional anesthesia, the perineural or interfascial administration of magnesium raises additional uncertainties regarding local tissue exposure, systemic absorption, and the possibility of dose-dependent neural effects that were not adequately captured in the included ESPB trials.

### 4.5. Proposed Core Outcome and Protocol Framework for Future Trials

To enable reproducible evaluation of magnesium as an ESPB adjuvant, future randomized trials should incorporate a standardized methodological framework.

1. Include a local anesthetic-only ESPB arm to isolate the incremental effect of magnesium.
2. Prespecify magnesium dosing (fixed or weight-based) with clear reporting of concentration, volume, and total administered dose.
3. Standardize the ESPB technique (level, laterality, ultrasound approach).
4. Adopt a core pain outcome set, including 24 h and 48 h pain intensity, using a validated continuous scale.
5. Report opioid consumption in morphine milligram equivalents with clearly defined rescue criteria.
6. Predefine adverse events and report incidence counts (n/N) by group with clinically relevant follow-up windows.
7. Ensure transparent allocation concealment, blinding procedures, and prespecified analysis plans.

Moreover, ESPB-specific trials are characterized by substantial heterogeneity in dosing strategies, technique, comparator choice, and outcome reporting. Unlike pooled analyses in classical peripheral nerve blocks, the current ESPB evidence base does not provide harmonized continuous pain outcomes or standardized opioid conversion metrics [21,42,43]. Therefore, conclusions drawn from pooled analyses of classical peripheral nerve blocks may not be directly applicable to the ESPB without dedicated ESPB-specific trials using harmonized protocols and outcome sets [21,42,43]. A dedicated ESPB-focused evidence base with standardized protocols and core outcome sets is required before conclusions drawn from other regional techniques can be applied to this fascial plane block.

Evidence from the broader regional anesthesia literature, including randomized trials of TAP block and paravertebral block, has suggested that perineural magnesium may prolong sensory block duration and reduce early postoperative pain [21,42,43,44,45,46,50,51,52]. However, these data derive from regional techniques with anatomical targets, diffusion patterns, and pharmacodynamic conditions that differ from those of the ESPB. Consequently, they should be interpreted as contextual rather than directly transferable evidence. The ESPB is a fascial plane block in which the injectate is deposited in an interfascial compartment rather than directly adjacent to a specific nerve. Therefore, the mechanisms through which magnesium may modulate analgesia in the ESPB likely involve interfascial diffusion and indirect neural effects rather than strictly perineural pharmacodynamics [11,13,53]. The ESPB is characterized by variable cranio-caudal and anterior diffusion patterns, with less predictable spread to ventral rami and paravertebral space [53,54,55]. The pharmacodynamic interaction between magnesium and local anesthetic in a fascial plane environment may, therefore, differ from that observed in conventional peripheral nerve blocks [54]. Accordingly, even if magnesium exerts beneficial effects in other regional anesthesia techniques, the fascial plane environment of the ESPB may attenuate, delay, or render less predictable any incremental pharmacodynamic interaction with local anesthetics. This interpretation remains biologically plausible but requires confirmation in adequately powered ESPB-specific randomized trials using standardized techniques and harmonized outcome reporting.

### 4.6. Strengths and Limitations of This Review

This review applied a predefined PICO framework, PRISMA 2020 methodology, structured narrative synthesis, and RoB 2 and GRADE assessments. A further strength of this review is the use of a predefined core comparison and anchor outcome to improve interpretability across heterogeneous ESPB trials. However, this review is limited by the small number of randomized trials and substantial clinical and methodological heterogeneity. Quantitative meta-analysis was not feasible due to inconsistent outcome definitions, heterogeneous reporting formats, incomplete adverse event tabulation, and limited availability of extractable continuous data. Potential publication bias cannot be excluded, and incomplete reporting in primary studies constrained secondary interpretation.

## 5. Conclusions

Current randomized evidence does not support the routine use of magnesium sulfate as an adjuvant in the ESPB. The available trials are few, heterogeneous, and provide very low certainty of evidence. Well-designed randomized trials using standardized ESPB techniques and harmonized outcome reporting are required before magnesium can be recommended in routine clinical practice.

## Figures and Tables

**Table 1:** Characteristics of included randomized controlled trials and ESPB protocols.

Study	Design/ Setting	Population (***n***)	ESPB Technique	Intervention (LA + MgSO_4_)	Comparator(s)	Outcomes Reported	Reported Safety Outcomes
Alansary et al., 2025 [28]	Prospective 3-arm RCT; corrective scoliosis surgery	Adolescents/young adults; *n* = 60	Bilateral bi-level ESPB (T5, T10)	Bupivacaine 0.125% (20 mL per injection per side per level) + MgSO_4_ 2 mg/kg	LA + dexmedetomidine; no ESPB control	Pain (NRS; AUC 0–48 h); morphine consumption (48 h, PCA)	Hemodynamics and adverse effects reported; others NR
Ahmed et al., 2022 [29]	Double-blind 3-arm RCT; PHN pain clinic	Adults; *n* = 75	ESPB level NR; unilateral	Bupivacaine 0.25% (20 mL) + MgSO_4_ 100 mg	Sham ESPB; LA alone	NRS-11 baseline, daily week 1, weeks 2–12; analgesic use (pregabalin, acetaminophen)	Safety outcomes NR
Refaat et al., 2023 [39]	Randomized single-blind comparative study; lumbar spine surgery	Adults; *n* = 52	Bilateral ESPB T10	Bupivacaine 0.25% (20 mL per side) + MgSO_4_ 125 mg per side	LA + dexmedetomidine	VAS in PACU and 2–12 h; rescue nalbuphine (VAS ≥ 4): total dose and time to first request	Hemodynamics reported; other safety outcomes NR
Aref et al., 2023 [30]	Prospective 3-arm randomized clinical study; pediatric inguinal hernia repair	Pediatric; *n* = 60	Unilateral ESPB T10	Bupivacaine 0.25% (0.5 mL/kg total volume) + MgSO_4_ (10% solution mixed 1:1)	LA alone; LA + dexamethasone	CHEOPS at 1–24 h; rescue paracetamol; time to first analgesic dose	Hemodynamics reported; others NR
Abdelbadie et al., 2022 [27]	Double-blind 2-arm RCT; posterior lumbar spinal fusion	Adults; *n* = 30	Bilateral ESPB; level NR	Bupivacaine 0.25% (20 mL per side) + MgSO_4_ 250 mg per side	LA alone	VAS (rest/movement) at 1–24 h; pethidine consumption (24 h); time to first request	Hemodynamics reported; others NR
El Sherif et al., 2022 [37]	Double-blind 3-arm RCT; modified radical mastectomy	Adult females; *n* = 60	Unilateral bi-level ESPB (T5, T7)	Levobupivacaine 0.25% (20 mL per level) + MgSO_4_ 2 mg/kg	LA + ketamine; LA alone	VAS PACU–48 h; morphine consumption (48 h); time to first PCA demand	Hemodynamics, PONV, sedation reported; no block complications
Elmaguid et al., 2025 [40]	Double-blind 3-arm RCT; modified radical mastectomy	Adult females; *n* = 75	ESPB T4; single level	Bupivacaine 0.25% (20 mL + adjuvant; total 22 mL) + MgSO_4_ 200 mg	LA + ketorolac; LA alone	VAS 0–24 h; chronic pain (BPI-SF) 1–6 months; opioid consumption (24 h); time to first request	Hemodynamics and PONV reported; pneumothorax 0%
Sachan et al., 2024 [38]	3-arm RCT; elective laparoscopic cholecystectomy	Adults; *n* = 60	Bilateral ESPB; level NR; 15 mL per side	Bupivacaine 0.25% (15 mL per side) + MgSO_4_ 500 mg total	LA + clonidine; LA alone	NRS immediately, 1, 6, 12, 24 h; rescue paracetamol	Hemodynamics and PONV reported; others NR

Abbreviations: AUC, area under the curve; BPI-SF, Brief Pain Inventory–Short Form; CHEOPS, Children’s Hospital of Eastern Ontario Pain Scale; ESPB, erector spinae plane block; IV, intravenous; LA, local anesthetic; MgSO_4_, magnesium sulfate; NRS, numeric rating scale; PACU, post-anesthesia care unit; PCA, patient-controlled analgesia; PHN, postherpetic neuralgia; PONV, postoperative nausea and vomiting; RCT, randomized controlled trial; VAS, visual analogue scale; NR, not reported.

**Table 2:** Outcome-specific risk of bias (RoB 2) judgments for included randomized trials.

Study	Pain Outcome: Overall RoB	Opioid/Rescue Outcome: Overall RoB	Key Rationale
Alansary et al., 2025 [28]	Some concerns	Some concerns	Randomization and allocation concealment reported. Patients and outcome assessors blinded; treating anesthetist not blinded. Prespecified analysis plan not clearly accessible.
Ahmed et al., 2022 [29]	Some concerns	Some concerns	Randomized double-blind design reported; allocation concealment and prespecified analysis plan not clearly described.
Refaat et al., 2023 [39]	Some concerns	Some concerns	Computer-generated block randomization reported, but single-blind design raises concern for deviations and measurement bias.
Aref et al., 2023 [30]	Some concerns	Some concerns	Computerized randomization reported; concealment and blinding procedures insufficiently detailed.
Abdelbadie et al., 2022 [27]	Some concerns	Some concerns	Randomized double-blind design with opaque envelopes and third-party preparation; protocol/statistical analysis plan not clearly accessible.
El Sherif et al., 2022 [37]	Low risk	Low risk	Computer-generated randomization, sealed opaque envelopes, and broad blinding support low risk across domains.
Elmaguid et al., 2025 [40]	Some concerns	Some concerns	Randomization and patient/assessor blinding described; some concern remains due to limited reporting-plan transparency.
Sachan et al., 2024 [38]	Some concerns	Some concerns	Randomized trial, but allocation concealment, blinding procedures, and reporting-plan details insufficiently described.

Abbreviations: RoB, risk of bias..

**Table 3:** Core subgroup outcome results: pain at 24 h and opioid/rescue analgesia for MgSO_4_ added to local anesthetic versus local anesthetic alone in adult postoperative surgery.

Study	Surgical Setting	Pain Scale and Reporting at 24 h	Pain Result at 24 h	Opioid/Rescue Analgesia Outcome	Direction of Effect
Abdelbadie et al., 2022 [27]	Posterior lumbar spinal fusion	VAS; median (range) at rest and movement	No clear between-group difference at 24 h	Pethidine consumption 24 h (mg, mean ± SD): 153 ± 13.6 (MgSO_4_) vs. 190 ± 12.8 (LA)	Lower opioid consumption reported in MgSO_4_ group
Elmaguid et al., 2025 [40]	Modified radical mastectomy	VAS; median (range) at rest and movement	No clear between-group difference at 24 h	Total opioid consumption 24 h (mg, mean ± SD): 9.92 ± 1.78 (MgSO_4_) vs. 20.0 ± 2.31 (LA)	Lower opioid consumption reported in MgSO_4_ group
Sachan et al., 2024 [38]	Laparoscopic cholecystectomy	NRS; dichotomized categories (NRS = 0 vs. ≥4)	Lower proportion of moderate-to-severe pain in MgSO_4_ group	Rescue tramadol 50 mg (patients receiving): 5/20 (MgSO_4_) vs. 20/20 (LA)	Fewer rescue events in MgSO_4_ group
El Sherif et al., 2022 [37]	Modified radical mastectomy	VAS; boxplots only (no extractable summary statistics)	Continuous values not extractable (boxplot-only reporting)	Morphine consumption 48 h (mg, mean ± SD): 7.00 ± 0.61 (MgSO_4_) vs. 14.40 ± 3.47 (LA)	Lower opioid consumption reported in MgSO_4_ group

Abbreviations: MgSO_4_, magnesium sulfate; LA, local anesthetic; NRS, numeric rating scale; VAS, visual analogue scale; SD, standard deviation. Note: Only participants in magnesium vs. local anesthetic-alone arms (*n* = 160) contributed to this comparison. Pain reporting at 24 h was heterogeneous and frequently non-extractable as continuous data. Opioid outcomes were reported using different drugs, time windows, and metrics, precluding quantitative pooling.

**Table 4:** Summary of protocol heterogeneity across included trials.

Domain of Heterogeneity	Observed Variability Across Trials
Clinical setting	Corrective scoliosis surgery; lumbar spine surgery; inguinal hernia repair (pediatric); laparoscopic cholecystectomy; modified radical mastectomy; postherpetic neuralgia
Patient population	Adults, pediatric patients, adolescents/young adults
ESPB vertebral level	T4, T5–T7, T5–T10, T10, or not reported
Injection laterality	Unilateral (surgical or affected side) or bilateral
Number of injection levels	Single-level or bi-level ESPB
Magnesium dosing strategy	Fixed-dose regimens (100–500 mg) or weight-based dosing (2 mg/kg)
Comparator interventions	Local anesthetic alone; dexmedetomidine; ketamine; clonidine; ketorolac; sham block; or no-block control
Outcome measurement and follow-up	Pain scales included NRS, VAS, and CHEOPS; follow-up ranged from immediate postoperative assessment to 6 months

Abbreviations: ESPB, erector spinae plane block; NRS, numeric rating scale; VAS, visual analogue scale; CHEOPS, Children’s Hospital of Eastern Ontario Pain Scale.

## Data Availability

Data extracted from published studies are contained within the article and Supplementary Materials. Further details are available from the corresponding author upon reasonable request.

## Supplementary Materials

The following supporting information can be downloaded at: https://www.mdpi.com/article/10.3390/life16050726/s1, Table S1. PICO framework and full electronic search strategies; Table S2. PRISMA 2020 checklist; Table S3. Full-text records assessed for eligibility, classification, and primary reason for non-inclusion as independent studies; Table S4. RoB 2 domain-level judgments by outcome; Table S5. Protocol variability matrix across included trials.

## Abbreviations

The following abbreviations are used in this manuscript:

AUC	Area under the curve
CHEOPS	Children’s Hospital of Eastern Ontario Pain Scale
ESPB	Erector spinae plane block
GRADE	Grading of Recommendations Assessment, Development, and Evaluation
MgSO_4_	Magnesium sulfate
NRS	Numeric rating scale
PACU	Post-anesthesia care unit
PCA	Patient-controlled analgesia
PHN	Postherpetic neuralgia
PONV	Postoperative nausea and vomiting
RCT	Randomized controlled trial
RoB	Risk of bias
VAS	Visual analogue scale

## References

[1] Forero M., Adhikary S.D., Lopez H., Tsui C., Chin K.J. (2016). The Erector Spinae Plane Block: A Novel Analgesic Technique in Thoracic Neuropathic Pain. Reg. Anesth. Pain Med..

[2] Oostvogels L., Weibel S., Meißner M., Kranke P., Meyer-Frießem C.H., Pogatzki-Zahn E., Schnabel A. (2024). Erector Spinae Plane Block for Postoperative Pain. Cochrane Database Syst. Rev..

[3] Kot P., Rodriguez P., Granell M., Cano B., Rovira L., Morales J., Broseta A., Andrés J.D. (2019). The Erector Spinae Plane Block: A Narrative Review. Korean J. Anesthesiol..

[4] Tsui B.C.H., Fonseca A., Munshey F., McFadyen G., Caruso T.J. (2019). The Erector Spinae Plane (ESP) Block: A Pooled Review of 242 Cases. J. Clin. Anesth..

[5] De Cassai A., Bonvicini D., Correale C., Sandei L., Tulgar S., Tonetti T. (2019). Erector Spinae Plane Block: A Systematic Qualitative Review. Minerva Anestesiol..

[6] Koo C.-H., Lee H.-T., Na H.-S., Ryu J.-H., Shin H.-J. (2022). Efficacy of Erector Spinae Plane Block for Analgesia in Thoracic Surgery: A Systematic Review and Meta-Analysis. J. Cardiothorac. Vasc. Anesth..

[7] Leong R.W., Tan E.S.J., Wong S.N., Tan K.H., Liu C.W. (2021). Efficacy of Erector Spinae Plane Block for Analgesia in Breast Surgery: A Systematic Review and Meta-Analysis. Anaesthesia.

[8] Cassai A.D., Bisi M., Nardelli M., Paiusco I., Talarico S., Fincati V., Dost B., Boscolo A., Navalesi P. (2025). Erector Spinae Plane Block for Postoperative Analgesia in Vertebral Surgery: An Umbrella Review of Systematic Reviews and Meta-Analyses. Korean J. Pain.

[9] Kendall M.C., Alves L., Traill L.L., De Oliveira G.S. (2020). The Effect of Ultrasound-Guided Erector Spinae Plane Block on Postsurgical Pain: A Meta-Analysis of Randomized Controlled Trials. BMC Anesthesiol..

[10] Liang X., Zhou W., Fan Y. (2021). Erector Spinae Plane Block for Spinal Surgery: A Systematic Review and Meta-Analysis. Korean J. Pain.

[11] Chin K.J., Lirk P., Hollmann M.W., Schwarz S.K.W. (2021). Mechanisms of Action of Fascial Plane Blocks: A Narrative Review. Reg. Anesth. Pain Med..

[12] Kirksey M.A., Haskins S.C., Cheng J., Liu S.S. (2015). Local Anesthetic Peripheral Nerve Block Adjuvants for Prolongation of Analgesia: A Systematic Qualitative Review. PLoS ONE.

[13] Luo J., Duan G., Huang H., Chen G. (2024). Research Status of Different Adjuvants on Nerve Block’s Effect. Pain Physician.

[14] Yang J., Zhang J.-W. (2022). Review of Benefits and Adverse Effects of the Most Commonly Used Local Anesthetic Adjuvants in Peripheral Nerve Blocks. J. Physiol. Pharmacol..

[15] Brummett C.M., Williams B.A. (2011). Additives to Local Anesthetics for Peripheral Nerve Blockade. Int. Anesthesiol. Clin..

[16] Fawcett W.J., Haxby E.J., Male D.A. (1999). Magnesium: Physiology and Pharmacology. Br. J. Anaesth..

[17] Herroeder S., Schönherr M.E., De Hert S.G., Hollmann M.W. (2011). Magnesium—Essentials for Anesthesiologists. Anesthesiology.

[18] Liu H.T., Hollmann M.W., Liu W.H., Hoenemann C.W., Durieux M.E. (2001). Modulation of NMDA Receptor Function by Ketamine and Magnesium: Part I. Anesth. Analg..

[19] Akutagawa T., Kitahata L.M., Saito H., Collins J.G., Katz J.D. (1984). Magnesium Enhances Local Anesthetic Nerve Block of Frog Sciatic Nerve. Anesth. Analg..

[20] Vastani N., Seifert B., Spahn D.R., Maurer K. (2013). Sensitivities of Rat Primary Sensory Afferent Nerves to Magnesium: Implications for Differential Nerve Blocks. Eur. J. Anaesthesiol..

[21] Li M., Jin S., Zhao X., Xu Z., Ni X., Zhang L., Liu Z. (2016). Does Magnesium Sulfate as an Adjuvant of Local Anesthetics Facilitate Better Effect of Perineural Nerve Blocks?: A Meta-Analysis of Randomized Controlled Trials. Clin. J. Pain.

[22] Onyeaka H., Adeola J., Xu R., Pappy A.L., Smucker M., Ufondu W., Osman M., Hasoon J., Orhurhu V. (2024). Intravenous Magnesium for the Management of Chronic Pain:An Updated Review of the Literature. Psychopharmacol. Bull..

[23] De Oliveira G.S., Castro-Alves L.J., Khan J.H., McCarthy R.J. (2013). Perioperative Systemic Magnesium to Minimize Postoperative Pain: A Meta-Analysis of Randomized Controlled Trials. Anesthesiology.

[24] Choi G.J., Kim Y.I., Koo Y.H., Oh H.-C., Kang H. (2021). Perioperative Magnesium for Postoperative Analgesia: An Umbrella Review of Systematic Reviews and Updated Meta-Analysis of Randomized Controlled Trials. J. Pers. Med..

[25] Pascual-Ramírez J., Gil-Trujillo S., Alcantarilla C. (2013). Intrathecal Magnesium as Analgesic Adjuvant for Spinal Anesthesia: A Meta-Analysis of Randomized Trials. Minerva Anestesiol..

[26] Albrecht E., Kirkham K.R., Liu S.S., Brull R. (2013). The Analgesic Efficacy and Safety of Neuraxial Magnesium Sulphate: A Quantitative Review. Anaesthesia.

[27] Abdelbadie M. (2022). Analgesic Efficacy of the Erector Spinae Plane Block Using Bupivacaine vs. Bupivacaine/Magnesium Sulphate in Patients Undergoing Lumbar Spine Surgery: A Randomized, Double-Blinded Comparative Study. Anaesth. Pain Intensive Care.

[28] Alansary A.M., Ali M.M., Elshafie M.A., Elbeialy M.A.K. (2025). Dexmedetomidine Versus Magnesium Sulfate in Ultrasound-Guided Bilateral Bi-Level Erector Spinae Plane Block in Corrective Scoliosis Surgery: A Randomized Controlled Clinical Trial. Clin. J. Pain.

[29] Ahmed S.A., Magdy A.A., Abdullah M.A., Albadry A.A. (2022). The Effect of Erector Spinae Plane Block with and Without Addition of Magnesium on Relief of Pain from Post-Herpetic Neuralgia. Pain Physician.

[30] Ahmed Mostafa Aref F., Abdelaziz N.M., Elazazzi H.M., Fahmy N.G. (2023). Effect of Addition of Different Additives: Magnesium Sulfate and Dexamethasone versus Plain Bupivacaine in Ultrasound-Guided Erector Spinae Plane Block in Pediatrics Undergoing Repair of Inguinal Hernia. Egypt. J. Anaesth..

[31] Page M.J., Moher D., Bossuyt P.M., Boutron I., Hoffmann T.C., Mulrow C.D., Shamseer L., Tetzlaff J.M., Akl E.A., Brennan S.E. (2021). PRISMA 2020 Explanation and Elaboration: Updated Guidance and Exemplars for Reporting Systematic Reviews. BMJ.

[32] Sterne J.A.C., Savović J., Page M.J., Elbers R.G., Blencowe N.S., Boutron I., Cates C.J., Cheng H.-Y., Corbett M.S., Eldridge S.M. (2019). RoB 2: A Revised Tool for Assessing Risk of Bias in Randomised Trials. BMJ.

[33] Guyatt G.H., Oxman A.D., Vist G.E., Kunz R., Falck-Ytter Y., Alonso-Coello P., Schünemann H.J., GRADE Working Group (2008). GRADE: An Emerging Consensus on Rating Quality of Evidence and Strength of Recommendations. BMJ.

[34] Page M.J., McKenzie J.E., Bossuyt P.M., Boutron I., Hoffmann T.C., Mulrow C.D., Shamseer L., Tetzlaff J.M., Akl E.A., Brennan S.E. (2021). The PRISMA 2020 Statement: An Updated Guideline for Reporting Systematic Reviews. BMJ.

[35] Cumpston M., Li T., Page M.J., Chandler J., Welch V.A., Higgins J.P., Thomas J. (2019). Updated Guidance for Trusted Systematic Reviews: A New Edition of the Cochrane Handbook for Systematic Reviews of Interventions. Cochrane Database Syst. Rev..

[36] Moher D., Liberati A., Tetzlaff J., Altman D.G. (2010). Preferred Reporting Items for Systematic Reviews and Meta-Analyses: The PRISMA Statement. Int. J. Surg..

[37] El Sherif F.A., Youssef H.A., Fares K.M., Mohamed S.A.-B., Ali A.R., Thabet A.M. (2022). Efficacy of Ketamine versus Magnesium Sulphate as Adjuvants to Levobupivacaine in Ultrasound Bilevel Erector Spinae Block in Breast Cancer Surgery (a Double-Blinded Randomized Controlled Study). Local Reg. Anesth..

[38] Sachan S., Radhakrishnan V., Paswan A.K. (2024). Comparative Evaluation of Bilateral Erector Spinae Plane Block Using Bupivacaine Combined with Magnesium Sulphate or Clonidine as an Adjuvant for Postoperative Analgesia in Laparoscopic Cholecystectomy—A Prospective Randomized Controlled Trial. Ain-Shams J. Anesthesiol..

[39] Refaat S.A., Abdelmageed W.M., Alwedeny H.M., Soliman E.H., Fouly M.A. (2023). Evaluation of the Effect of Dexmedetomidine versus Magnesium Sulphate as an Adjuvant to Bupivacaine in Ultrasound Guided Erector Spinae Block; a Prospective Randomized Trial. Anaesth. Pain Intensive Care.

[40] Elmaguid M.A., Youssef M.Y., Hegazy M.A., Shams T. (2025). Analgesic Efficacy of Magnesium Sulphate versus Ketorolac When Added to Bupivacaine in Erector Spinae Plane Block for Acute and Chronic Postmastectomy Pain. Egypt. J. Hosp. Med..

[41] Dworkin R.H., Turk D.C., Farrar J.T., Haythornthwaite J.A., Jensen M.P., Katz N.P., Kerns R.D., Stucki G., Allen R.R., Bellamy N. (2005). Core Outcome Measures for Chronic Pain Clinical Trials: IMMPACT Recommendations. Pain.

[42] Zeng J., Chen Q., Yu C., Zhou J., Yang B. (2021). The Use of Magnesium Sulfate and Peripheral Nerve Blocks: An Updated Meta-Analysis and Systematic Review. Clin. J. Pain.

[43] Pal S., Kainth R.K., Malhotra R., Banerjee A., Banerjee R. (2025). Magnesium in Brachial Plexus Blocks: A Meta-Analysis, Meta Regression, and Trial Sequential Analysis. Indian J. Anaesth..

[44] Lee A.R., Yi H., Chung I.S., Ko J.S., Ahn H.J., Gwak M.S., Choi D.H., Choi S.J. (2012). Magnesium Added to Bupivacaine Prolongs the Duration of Analgesia after Interscalene Nerve Block. Can. J. Anaesth..

[45] Ammar A.S., Mahmoud K.M. (2014). Does the Addition of Magnesium to Bupivacaine Improve Postoperative Analgesia of Ultrasound-Guided Thoracic Paravertebral Block in Patients Undergoing Thoracic Surgery?. J. Anesth..

[46] ELShamaa H.A., Ibrahim M., Eldesuky H.L. (2014). Magnesium Sulfate in Femoral Nerve Block, Does Postoperative Analgesia Differ? A Comparative Study. Egypt. J. Anaesth..

[47] Zhang Y., Huang Y., Li J. (2025). Adverse Drug Events Observed with Intrathecal Magnesium Sulfate as an Adjuvant to Bupivacaine for Spinal Anesthesia in Patients Undergoing Elective Cesarean Section: A Meta-Analysis. BMC Pharmacol. Toxicol..

[48] Ozdogan L., Sastim H., Ornek D., Postaci A., Ayerden T., Dikmen B. (2013). Neurotoxic Effects of Intrathecal Magnesium Sulphate. Braz. J. Anesthesiol..

[49] Morrison A.P., Hunter J.M., Halpern S.H., Banerjee A. (2013). Effect of Intrathecal Magnesium in the Presence or Absence of Local Anaesthetic with and without Lipophilic Opioids: A Systematic Review and Meta-Analysis. Br. J. Anaesth..

[50] Abd-Elsalam K.A., Fares K.M., Mohamed M.A., Mohamed M.F., El-Rahman A.M.A., Tohamy M.M. (2017). Efficacy of Magnesium Sulfate Added to Local Anesthetic in a Transversus Abdominis Plane Block for Analgesia Following Total Abdominal Hysterectomy: A Randomized Trial. Pain Physician.

[51] Imani F., Rahimzadeh P., Faiz H.-R., Abdullahzadeh-Baghaei A. (2018). An Evaluation of the Adding Magnesium Sulfate to Ropivacaine on Ultrasound-Guided Transverse Abdominis Plane Block After Abdominal Hysterectomy. Anesth. Pain Med..

[52] Shambhavi T., Das S., Senapati L.K., Padhi P.P. (2023). Comparative Evaluation of Bupivacaine with Magnesium Sulphate and Dexamethasone as Adjuvants in Ultrasound-Guided Transversus Abdominis Plane Block for Open Unilateral Inguinal Hernia Surgeries: A Randomised Controlled Trial. Indian J. Anaesth..

[53] Adhikary S.D., Bernard S., Lopez H., Chin K.J. (2018). Erector Spinae Plane Block Versus Retrolaminar Block: A Magnetic Resonance Imaging and Anatomical Study. Reg. Anesth. Pain Med..

[54] Ivanusic J., Konishi Y., Barrington M.J. (2018). A Cadaveric Study Investigating the Mechanism of Action of Erector Spinae Blockade. Reg. Anesth. Pain Med..

[55] Bonvicini D., Boscolo-Berto R., De Cassai A., Negrello M., Macchi V., Tiberio I., Boscolo A., De Caro R., Porzionato A. (2021). Anatomical Basis of Erector Spinae Plane Block: A Dissection and Histotopographic Pilot Study. J. Anesth..

