# Supplementary material for: Magnesium Sulfate as an Adjuvant to Local Anesthetic in Erector Spinae Plane Block: A Systematic Review of Randomized Controlled Trials
Source: Life (Basel). 2026 Apr 25;16(5):726. doi: 10.3390/life16050726 (PMC13208695; doi:10.3390/life16050726)
Supplement: Supplementary file 1 [file life-16-00726-s001.zip › Supplementary Table S1. PICO framework and full electronic search strategies.pdf]

Supplementary Table S1. PICO and search strategy

Review question

In patients undergoing surgery or treated for pain with ultrasound-guided ESPB, does perineural magnesium sulfate added to local anesthetics improve analgesic outcomes and safety compared with ESPB without magnesium or compared with ESPB performed with other single perineural adjuvants, based on randomized controlled trials?

PICO framework

Population: Adults and children receiving ultrasound-guided ESPB for surgical procedures or pain management (acute or neuropathic pain).

Intervention: Perineural magnesium sulfate ($MgSO_4$) added to any local anesthetic solution for ESPB.

Comparators: 1) The same ESPB local anesthetic without $MgSO_4$ (placebo/saline or no adjuvant); and/or 2) The same ESPB local anesthetic plus another single perineural adjuvant (e.g., dexmedetomidine, ketamine, dexamethasone), analyzed as a separate comparison.

Outcomes: Primary: pain intensity (VAS/NRS; FLACC/CHEOPS where applicable) at 12–24 hours after surgery/block. Secondary: pain at other time points; opioid consumption within 24 h (or 48 h if reported), converted to morphine milligram equivalents when possible; time to first rescue analgesia; block characteristics (onset/duration) if reported; adverse events (hypotension, bradycardia, PONV, sedation, respiratory depression, neurological deficits, local anesthetic systemic toxicity, magnesium toxicity).

Study design: Randomized controlled trials (parallel-group). Cluster/crossover RCTs will be included if methodologically appropriate for outcomes.

Eligibility criteria

Inclusion criteria:

• Randomized controlled trials evaluating ultrasound-guided ESPB with perineural $MgSO_4$ added to a local anesthetic.

• Comparator: ESPB with the same local anesthetic alone (no $MgSO_4$) and/or ESPB with the same local anesthetic plus another single adjuvant (head-to-head).

• Adult or pediatric populations; perioperative or pain clinic settings; any language; full-text publications.

Exclusion criteria:

• Non-randomized studies, case reports/series, narrative reviews, editorials, letters without primary RCT data.

• Conference abstracts without accessible full-text publication.

• Trial registry records without a corresponding full-text peer-reviewed publication.

• Comparisons where the technique differs between groups (e.g., ESPB+$MgSO_4$ vs a different regional block), because the effect of $MgSO_4$ cannot be attributed within ESPB.

Information sources and search limits

Databases from inception to the date of the new search execution: MEDLINE (PubMed), Embase, Scopus, and Cochrane CENTRAL; studies previously identified via citation search will be included through manual searching and reported in PRISMA as 'other methods'.

No language restrictions. No date restrictions other than the search end date.

Search strategies:

MEDLINE (PubMed)

("Erector Spinae Plane Block"[Title/Abstract] OR "erector spinae plane block"[Title/Abstract] OR ESPB[Title/Abstract] OR (erector spinae[Title/Abstract] AND (block*[Title/Abstract] OR plane[Title/Abstract]))) AND (magnesium[Title/Abstract] OR "magnesium sulfate"[Title/Abstract] OR "magnesium sulphate"[Title/Abstract] OR MgSO4[Title/Abstract]) AND (randomized[Title/Abstract] OR randomised[Title/Abstract] OR randomly[Title/Abstract] OR trial[Title/Abstract] OR "controlled trial"[Title/Abstract])

Embase

(('erector spinae plane block'/exp OR 'erector spinae plane block':ti,ab OR ESPB:ti,ab OR (erector spinae:ti,ab AND (block*:ti,ab OR plane:ti,ab))) AND ('magnesium sulfate'/exp OR magnesium:ti,ab OR 'magnesium sulfate':ti,ab OR 'magnesium sulphate':ti,ab OR MgSO4:ti,ab) AND ('randomized controlled trial'/exp OR random*:ti,ab OR trial:ti,ab OR placebo:ti,ab))

Cochrane CENTRAL

("erector spinae" AND (block OR plane OR ESPB)) AND (magnesium OR "magnesium sulfate" OR "magnesium sulphate" OR MgSO4)

Scopus

TITLE-ABS-KEY(("erector spinae" W/2 (block* OR plane) OR ESPB) AND (magnesium OR "magnesium sulfate" OR "magnesium sulphate" OR MgSO4) AND (random* OR trial OR placebo))
