# Supplementary material for: Magnesium Sulfate as an Adjuvant to Local Anesthetic in Erector Spinae Plane Block: A Systematic Review of Randomized Controlled Trials
Source: Life (Basel). 2026 Apr 25;16(5):726. doi: 10.3390/life16050726 (PMC13208695; doi:10.3390/life16050726)
Supplement: Supplementary file 1 [file life-16-00726-s001.zip › Supplementary Table S2. Prisma 2020 checklist.pdf]

**Supplementary Table S2**. Prisma 2020 checklist

| Section and Topic | Item # | Checklist item | Location where item is reported |
|---|---|---|---|
| **TITLE** | 1 | Identify the report as a systematic review. | Title |
| **ABSTRACT** | 2 | See the PRISMA 2020 for Abstracts checklist. | Abstract |
| **INTRODUCTION** | 3 | Describe the rationale for the review in the context of existing knowledge. | Introduction |
| **INTRODUCTION** | 4 | Provide an explicit statement of the objective(s) or question(s) the review addresses. | Introduction (final paragraph) |
| **METHODS** | 5 | Specify the inclusion and exclusion criteria for the review and how studies were grouped for the syntheses. | Methods – Eligibility criteria |
| **METHODS** | 6 | Specify all databases, registers, websites, organisations, reference lists and other sources searched or consulted to identify studies. Specify the date when each source was last searched or consulted. | Methods – Information sources |
| **METHODS** | 7 | Present the full search strategies for all databases, registers and websites, including any filters and limits used. | Supplementary Table S1 |
| **METHODS** | 8 | Specify the methods | Methods – Study |

| | | | |
|---|---|---|---|
| | | used to decide whether a study met the inclusion criteria of the review, including how many reviewers screened each record and report retrieved, whether they worked independently, and if applicable, details of automation tools used in the process. | selection |
| **METHODS** | 9 | Specify the methods used to collect data from reports, including how many reviewers collected data from each report, whether they worked independently, any processes for obtaining or confirming data from study investigators, and if applicable, details of automation tools used in the process. | Methods – Data extraction |
| **METHODS** | 10a | List and define all outcomes for which data were sought. | Methods – Eligibility criteria; Data extraction; Synthesis methods |
| **METHODS** | 10b | List and define all other variables for which data were sought. | Methods – Data extraction |
| **METHODS** | 11 | Specify the methods used to assess risk of bias in the included studies. | Methods – Risk of bias assessment |
| **METHODS** | 12 | Specify for each outcome the effect measure(s) used in the synthesis or presentation of | Methods – Synthesis methods |

| | | | |
|---|---|---|---|
| | | results. | |
| **METHODS** | 13a | Describe the processes used to decide which studies were eligible for each synthesis. | Methods – Synthesis methods |
| **METHODS** | 13b | Describe any methods required to prepare the data for presentation or synthesis. | Methods – Synthesis methods |
| **METHODS** | 13c | Describe any methods used to tabulate or visually display results of individual studies and syntheses. | Methods – Synthesis methods; Results – Tables 1–4 and Figures 1–2 |
| **METHODS** | 13d | Describe any methods used to synthesize results and provide a rationale for the choice(s). | Methods ‑ Synthesis methods |
| **METHODS** | 13e | Describe any methods used to explore possible causes of heterogeneity among study results. | Results – Protocol variability analysis |
| **METHODS** | 13f | Describe any sensitivity analyses conducted to assess robustness of the synthesized results. | Not performed |
| **METHODS** | 14 | Describe any methods used to assess risk of bias due to missing results in a synthesis. | Methods – Certainty of evidence |
| **METHODS** | 15 | Describe any methods used to assess certainty in the body of evidence for an outcome. | Methods – Certainty of evidence |
| **RESULTS** | 16a | Describe the results of the search and selection process, | Results – Study selection; Figure 1 |

| | | | |
|---|---|---|---|
| | | ideally using a flow diagram. | |
| **RESULTS** | 16b | Cite studies that might appear to meet the inclusion criteria but were excluded, and explain why. | Results – Study selection |
| **RESULTS** | 17 | Cite each included study and present its characteristics. | Results – Study characteristics; Table 1 |
| **RESULTS** | 18 | Present assessments of risk of bias for each included study. | Results – Risk of bias; Table 2 |
| **RESULTS** | 19 | For all outcomes, present summary statistics and effect estimates for each study. | Results – Sections 3.4–3.8 |
| **RESULTS** | 20a | For each synthesis, summarise the characteristics and risk of bias among contributing studies. | Results – Core subgroup analysis |
| **RESULTS** | 20b | Present results of all statistical syntheses conducted. | Narrative synthesis (no meta-analysis performed) |
| **RESULTS** | 20c | Present results of investigations of possible causes of heterogeneity among study results. | Results – Protocol variability analysis |
| **RESULTS** | 20d | Present results of all sensitivity analyses conducted to assess robustness of the synthesized results. | Not performed |
| **RESULTS** | 21 | Present assessments of risk of bias due to missing results. | Discussion – Limitations |
| **RESULTS** | 22 | Present assessments of certainty in the body of evidence for each outcome assessed. | Results – Certainty of evidence |

| | | | |
|---|---|---|---|
| **DISCUSSION** | 23a | Provide a general interpretation of the results in the context of other evidence. | Discussion |
| **DISCUSSION** | 23b | Discuss any limitations of the evidence included in the review. | Discussion – Strengths and limitations |
| **DISCUSSION** | 23c | Discuss any limitations of the review processes used. | Discussion – Strengths and limitations |
| **DISCUSSION** | 23d | Discuss implications of the results for practice, policy, and future research. | Discussion – Implications |
| **OTHER INFORMATION** | 24a | Provide registration information for the review. | Methods – Protocol and reporting standards |
| **OTHER INFORMATION** | 24b | Indicate where the review protocol can be accessed. | Methods – Protocol and reporting standards; PROSPERO record (CRD420261343098) |
| **OTHER INFORMATION** | 24c | Describe and explain any amendments to information provided at registration. | Methods – Protocol and reporting standards |
| **OTHER INFORMATION** | 25 | Describe sources of financial or non-financial support for the review. | Funding |
| **OTHER INFORMATION** | 26 | Declare any competing interests of review authors. | Conflicts of Interest |
| **OTHER INFORMATION** | 27 | Report which materials are publicly available and where they can be found. | Data Availability Statement |
