# Supplementary material for: Magnesium Sulfate as an Adjuvant to Local Anesthetic in Erector Spinae Plane Block: A Systematic Review of Randomized Controlled Trials
Source: Life (Basel). 2026 Apr 25;16(5):726. doi: 10.3390/life16050726 (PMC13208695; doi:10.3390/life16050726)
Supplement: Supplementary file 1 [file life-16-00726-s001.zip › Supplementary Table S3. Full-text records assessed for eligibility and reasons for exclusion.pdf]

Table S3. Full-text records assessed for eligibility, classification, and primary reason for non-inclusion as independent studies

Record	Source	Classification	Primary reason for non-inclusion as an independent study
NCT06501079	ClinicalTrials.gov	Excluded full-text record	No peer-reviewed full-text publication available
NCT05855798	ClinicalTrials.gov	Excluded full-text record	No peer-reviewed full-text publication available
NCT07139522	ClinicalTrials.gov	Excluded full-text record	No peer-reviewed full-text publication available
NCT04732390	ClinicalTrials.gov	Excluded full-text record	No peer-reviewed full-text publication available
PACTR202502528583311	PACTR	Excluded full-text record	No peer-reviewed full-text publication available
NCT05851768	ClinicalTrials.gov	Excluded full-text record	No peer-reviewed full-text publication available
CTRI/2025/06/089542	CTRI	Excluded full-text record	No comparator ESPB without magnesium
CTRI/2024/10/074814	CTRI	Excluded full-text record	No peer-reviewed full-text publication available
CTRI/2024/12/077711	CTRI	Excluded full-text record	No peer-reviewed full-text publication available
NCT04732364	ClinicalTrials.gov	Excluded full-text record	No peer-reviewed full-text publication available
NCT05976464	ClinicalTrials.gov	Companion record of included study (Trial registry record)	Not an independent study

NCT04275661	ClinicalTrials.gov	Companion record of included study (Trial registry record)	Not an independent study
Hussien et al., 2024	QJM: An International Journal of Medicine	Companion record of included study (Conference abstract)	Not an independent study

Supplementary reference details for records listed in Table S3

A. Full-text excluded records

1. **ClinicalTrials.gov Identifier: NCT06501079.**
The Efficacy of Dexamethasone Versus Magnesium Sulphate Added to Bupivacaine Used in Erector Spinae Plane Block for Perioperative Pain Control in Patients Undergoing Unilateral Nephrectomy.
ClinicalTrials.gov registry record. 2024.

2. **ClinicalTrials.gov Identifier: NCT05855798.**
Comparison of Analgesic Effects of Magnesium Sulphate and Ketamine Added to 0.5% Bupivacaine for Erector Spinae Plane Block in Patients Undergoing Video-assisted Thoracoscopic Surgery.
ClinicalTrials.gov registry record. 2023.

3. **ClinicalTrials.gov Identifier: NCT07139522.**
Dexamethasone Versus Magnesium Sulphate as an Adjuvant to Bupivacaine in Ultrasound Guided Erector Spinae Plane Block for Postoperative Analgesia in Elective Caesarean Section Under Spinal Anesthesia.
ClinicalTrials.gov registry record; recruiting.

4. **ClinicalTrials.gov Identifier: NCT04732390.**
Analgesic Efficacy of Magnesium Sulphate as an Adjuvant to Levobupivacaine in Erector Spinae Block for Acute Pain Management in Modified Radical Mastectomy.
ClinicalTrials.gov registry record; status listed as unknown.

5. **Pan African Clinical Trials Registry (PACTR). Main ID: PACTR202502528583311.**
Public title: *Erector spinae ketamine versus magnesium sulphate in thoracotomy.*
Scientific title: *Ketamine versus Magnesium Sulphate in Erector Spinae Plane Block for Postoperative Analgesia Following Thoracotomy (a Double-Blinded Randomized Controlled Study).*
Date of registration: 05/02/2025; recruitment status: Pending.

6. **ClinicalTrials.gov Identifier: NCT05851768.**
Efficacy of Dexmedetomidine VS Magnesium Sulphate With Bupivacaine in Erector Spinae Block for Thoracotomy Pain.
ClinicalTrials.gov registry record; status listed as unknown.

7. **Clinical Trials Registry–India (CTRI). Main ID: CTRI/2025/06/089542.**
Public title: *Comparison of two pain relieving techniques for breast cancer surgeries.*
Scientific title: *COMPARISON OF ULTRASOUND GUIDED ERECTOR SPINAE PLANE BLOCK VERSUS SERRATUS ANTERIOR PLANE BLOCK IN ONCOLOGY BREAST SURGERIES- A RANDOMISED CONTROL STUDY - nil.*
Date of registration: 25-06-2025; recruitment status: Open to Recruitment.

8. **Clinical Trials Registry–India (CTRI). Main ID: CTRI/2024/10/074814.**
Public title: *Comparing Magnesium Sulfate and Dexmedetomidine for Better Recovery in Breast Cancer Surgery with Local Anesthesia.*
Scientific title: *To Compare Quality of Recovery with Magnesiumsulphate versus Dexmedetomidine as Adjuvant to Levobupivacaine in Erector Spinae Plane Block in Modified Radical Mastectomy -Randomized Controlled Study - nil.*
Date of registration: 07-10-2024; recruitment status: Not Yet Recruiting.

9. **Clinical Trials Registry–India (CTRI). Main ID: CTRI/2024/12/077711.**
Public title: *A clinical trial to study the effects of two drugs, Adenosine and Magnesium Sulphate for control of pain after Gall bladder surgery.*
Scientific title: *Adenosine vs Magnesium Sulphate as Adjuvants in Erector Spinae Plane block for post operative*

analgesia in patients undergoing Laproscopic Cholecystectomy : A Randomized Clinical Trial - Nil.
Date of registration: 05-12-2024; recruitment status: Not Yet Recruiting.

10. **ClinicalTrials.gov Identifier: NCT04732364.**
Analgesic Efficacy of Ultrasound Guided Erector Spinae Block for Modified Radical Mastectomy.
ClinicalTrials.gov registry record; status listed as unknown.

B. Companion records of included studies

11. **ClinicalTrials.gov Identifier: NCT05976464.**
Efficacy of Magnesium Sulfate as an Adjuvant to Local Anesthetic in Erector Spinae Plane Block for Post-operative Analgesia After Modified Radical Mastectomy.
ClinicalTrials.gov registry record.

Companion record of an included study; not an independent study.

12. **ClinicalTrials.gov Identifier: NCT04275661.**
Ketamine Versus Magnesium Sulphate as Adjuvants for ESPB in Breast Cancer Surgery.
ClinicalTrials.gov registry record; completed.

Companion record of an included study; not an independent study.

13. Ehab Hussien Soliman Hussien, Waleed Mohamed Abdelmageed, Hadeel Magdy Alwedeny, Sameh Ahmed Refaat, Mohammed Abdelmoneim Fouly, Ultrasound Guided Erector Spinae Block Using Bupivacaine – Magnesium Sulphate versus Bupivacaine – Dexmedetomidine for Postoperative Analgesia in Lumbar Spine Surgeries, QJM: An International Journal of Medicine, Volume 117, Issue Supplement_1, June 2024, hcae070.083, https://doi.org/10.1093/qjmed/hcae070.083

Companion conference abstract of an included study; not an independent study.
