# Supplementary material for: Magnesium Sulfate as an Adjuvant to Local Anesthetic in Erector Spinae Plane Block: A Systematic Review of Randomized Controlled Trials
Source: Life (Basel). 2026 Apr 25;16(5):726. doi: 10.3390/life16050726 (PMC13208695; doi:10.3390/life16050726)
Supplement: Supplementary file 1 [file life-16-00726-s001.zip › Supplementary Table S4. RoB 2 domain-level judgments by outcome.pdf]

Supplementary Table S4. RoB 2 domain-level judgments by outcome.

Study	Outcome	Randomization process	Deviations from intended interventions	Missing outcome data	Measurement of the outcome	Selection of the reported result	Overall judgment
**Alansary et al., 2025 [28]**	Pain	Low	Some concerns	Low	Low	Some concerns	Some concerns
**Alansary et al., 2025 [28]**	Opioid/rescue	Low	Some concerns	Low	Low	Some concerns	Some concerns
**Ahmed et al., 2022 [29]**	Pain	Some concerns	Low	Low	Low	Some concerns	Some concerns
**Ahmed et al., 2022 [29]**	Opioid/rescue	Some concerns	Low	Low	Low	Some concerns	Some concerns
**Refaat et al., 2023 [39]**	Pain	Low	Some concerns	Low	Some concerns	Some concerns	Some concerns
**Refaat et al., 2023 [39]**	Opioid/rescue	Low	Some concerns	Low	Some concerns	Some concerns	Some concerns
**Aref et al., 2023 [30]**	Pain	Some concerns	Low	Low	Low	Some concerns	Some concerns
**Aref et al., 2023 [30]**	Opioid/rescue	Some concerns	Low	Low	Low	Some concerns	Some concerns
**Abdelbadie et al., 2022 [27]**	Pain	Low	Low	Low	Low	Some concerns	Some concerns
**Abdelbadie et al., 2022 [27]**	Opioid/rescue	Low	Low	Low	Low	Some concerns	Some concerns
**El Sherif et al., 2022 [37]**	Pain	Low	Low	Low	Low	Low	Low risk
**El Sherif et al., 2022 [37]**	Opioid/rescue	Low	Low	Low	Low	Low	Low risk
**Elmaguid et al., 2025 [40]**	Pain	Low	Low	Low	Low	Some concerns	Some concerns
**Elmaguid et al., 2025 [40]**	Opioid/rescue	Low	Low	Low	Low	Some concerns	Some concerns
**Sachan et al., 2024 [38]**	Pain	Some concerns	Low	Low	Low	Some concerns	Some concerns
**Sachan et al., 2024 [38]**	Opioid/rescue	Some concerns	Low	Low	Low	Some concerns	Some concerns

Abbreviations: RoB, risk of bias.

Note: Judgments were performed outcome-specifically (pain intensity and opioid/rescue outcomes) following RoB 2 guidance; overall judgment reflects the highest level of concern across domains.
