# Supplementary material for: Magnesium Sulfate as an Adjuvant to Local Anesthetic in Erector Spinae Plane Block: A Systematic Review of Randomized Controlled Trials
Source: Life (Basel). 2026 Apr 25;16(5):726. doi: 10.3390/life16050726 (PMC13208695; doi:10.3390/life16050726)
Supplement: Supplementary file 1 [file life-16-00726-s001.zip › Supplementary Table S5. Protocol variability matrix across included trials.pdf]

Supplementary Table S3. Protocol Variability Matrix Across Included Randomized Controlled Trials

Study ID	Clinical context	ESPB technique	Local anesthetic	$MgSO_4$ dose strategy	Comparator(s)	Pain outcome reporting	Opioid/rescue reporting	Safety reporting
Alansary et al., 2025 [28]	Prospective 3-arm RCT; corrective scoliosis surgery	Bi-level T5 & T10; bilateral	Bupivacaine 0.125%; 20 mL per injection (per side per level)	2 mg/kg per side per injection	Dexmedetomidine 0.25 µg/kg per side per injection; and no-ESPB control	NRS; 0–48 h summarized as time-weighted average (AUC)	Morphine consumption over 48 h (PCA described)	Hemodynamics and adverse effects reported; others NR/unclear
Ahmed et al., 2022 [29]	Double-blind 3-arm RCT; PHN pain clinic	Level NR (based on dermatomes); unilateral (affected side)	Bupivacaine 0.25%; 20 mL	100 mg (fixed dose)	Sham ESPB (2 mL normal saline); and bupivacaine-only ESPB	NRS-11; baseline; daily week 1; weeks 2–12	Analgesic consumption: pregabalin and acetaminophen (no opioid consumption outcome)	Safety outcomes NR
Refaat et al., 2023 [39]	Randomized single-blind comparative study; lumbar spine surgeries	T10; bilateral single-level	Bupivacaine 0.25%; 20 mL per side (total injectate 25 mL per side)	125 mg per side (fixed)	Dexmedetomidine 1 µg/kg (added per side)	VAS; PACU and 2–12 h	Rescue nalbuphine (VAS ≥4): total dose and time to first request (0–12 h)	Hemodynamics reported; PONV/sedation/complications NR
Aref et al., 2023 [30]	Prospective 3-arm randomized clinical study; pediatric inguinal hernia repair	T10; unilateral (hernia side)	Bupivacaine 0.25%; 0.5 mL/kg (total injectate volume)	NR in mg: $MgSO_4$ 10% mixed 1:1 with bupivacaine within 0.5 mL/kg	Bupivacaine-only; and dexamethasone 0.2 mg/kg + bupivacaine	CHEOPS; 1, 2, 4, 6, 8, 16, 24 h	Rescue paracetamol; time to first analgesic dose (criteria NR)	Hemodynamics reported; others NR
Abdelbadie et al., 2022	Double-blind 2-arm RCT;	Level NR;	Bupivacaine 0.25%; 20 mL	250 mg per	Bupivacaine-only	VAS (rest & movement); 1,	Pethidine consumption	Hemodynamics reported; PONV/sedation/complications

[27]	posterior lumbar spinal fusion	bilateral	per side	side (fixed)	ESPB	6, 12, 24 h	(24 h) and time to first request	NR
El Sherif et al., 2022 [37]	Double-blind 3-arm RCT; modified radical mastectomy	Bi-level T5 & T7; unilateral (surgical side)	Levobupivacaine 0.25%; 20 mL per level (2 injections)	2 mg/kg (weight-based)	Ketamine 2 mg/kg; and levobupivacaine-only	VAS (rest & movement); PACU to 48 h (multiple timepoints)	Total morphine consumption over 48 h (PCA + rescue); time to first PCA demand	Hemodynamics, PONV and sedation reported; block complications none reported
Elmaguid et al., 2025 [40]	Double-blind 3-arm RCT; modified radical mastectomy	T4; single-level (laterality not explicitly stated; surgical context suggests unilateral)	Bupivacaine 0.25%; 20 mL + 2 mL adjuvant (total 22 mL)	200 mg (2 mL of 10% $MgSO_4$)	Ketorolac 30 mg; and control (2 mL saline)	VAS (rest & movement); 0, 2, 4, 6, 12, 18, 24 h; chronic pain: BPI-SF 1–6 months	Total opioid consumption over 24 h (reported as morphine in abstract); time to first analgesic request	Hemodynamics and PONV reported; pneumothorax 0% in all groups
Sachan et al., 2024 [38]	3-arm RCT; elective laparoscopic cholecystectomy	Level NR; bilateral (15 mL per side)	Bupivacaine 0.25%; 15 mL per side	500 mg total (dose per side not explicitly stated)	Clonidine 1.5 µg/kg; and bupivacaine-only	NRS; immediate; 1, 6, 12, 24 h (rest/movement NR)	Rescue analgesia: paracetamol 1 g; opioid consumption not clearly presented	Hemodynamics and PONV reported; others NR

Abbreviations: ESPB, erector spinae plane block; NRS, numeric rating scale; VAS, visual analogue scale; CHEOPS, Children's Hospital of Eastern Ontario Pain Scale.
